# The Hepatic Monocarboxylate Transporter 1 (MCT1) Contributes to the Regulation of Food Anticipation in Mice

**DOI:** 10.3389/fphys.2021.665476

**Published:** 2021-04-14

**Authors:** Tomaz Martini, Jürgen A. Ripperger, Rohit Chavan, Michael Stumpe, Citlalli Netzahualcoyotzi, Luc Pellerin, Urs Albrecht

**Affiliations:** ^1^Department of Biology, Faculty of Science and Medicine, University of Fribourg, Fribourg, Switzerland; ^2^Department of Physiology, University of Lausanne, Lausanne, Switzerland; ^3^Faculty of Health Sciences, Anahuac University, Naucalpan de Juárez, Mexico; ^4^Inserm U1082, University of Poitiers, Poitiers, France

**Keywords:** hydroxybutyric acid, food-anticipatory activity, restricted feeding, circadian rhythms, period 2, *Per2*, *Slc16a1*, ketone bodies

## Abstract

Daily recurring events can be predicted by animals based on their internal circadian timing system. However, independently from the suprachiasmatic nuclei (SCN), the central pacemaker of the circadian system in mammals, restriction of food access to a particular time of day elicits food anticipatory activity (FAA). This suggests an involvement of other central and/or peripheral clocks as well as metabolic signals in this behavior. One of the metabolic signals that is important for FAA under combined caloric and temporal food restriction is β-hydroxybutyrate (βOHB). Here we show that the monocarboxylate transporter 1 (*Mct1*), which transports ketone bodies such as βOHB across membranes of various cell types, is involved in FAA. In particular, we show that lack of the *Mct1* gene in the liver, but not in neuronal or glial cells, reduces FAA in mice. This is associated with a reduction of βOHB levels in the blood. Our observations suggest an important role of ketone bodies and its transporter *Mct1* in FAA under caloric and temporal food restriction.

## Introduction

Reproduction, feeding and avoidance of predators are key to survival of animals. These three existential processes require precise time-keeping to determine when an animal rests or seeks food. In mice, nocturnal activity and feeding, as well as daytime rest are determined by an internal time-keeping system that is entrained by light (Dibner et al., 2010). As a consequence, mice prefer to eat during the night (Challet, 2019). However, by restricting food to the day or inactivity phase, as in the case of daytime-restricted feeding (RF), mice will adapt and show an increase in activity and internal body temperature before the recurrent food availability (Mistlberger, 2011; Challet, 2019). This adaptation is commonly referred to as food anticipation (FA). FA is a response of animals to food availability at a certain time-point or lack of food during the rest of the day. Interestingly, this anticipation of feeding time persists for at least 3 days after the food is withdrawn, and it can reappear during food deprivation tests after a week or more of *ad libitum* feeding (Coleman et al., 1982; Rosenwasser et al., 1984). Hence, there is a time-keeping mechanism driving this behavior, which operates once a day (Blum et al., 2012).

Due to its robustness of onset during constant lighting conditions, early on it was speculated that FA is linked to the circadian clock system. This is further supported by the fact that the repeated feeding, required for the adaptation, needs to be performed in increments within a certain range, suggesting a biological limitation of the cycle length. Essentially every organ has the ability to create circadian rhythms (Stokkan et al., 2001). However, peripheral circadian clocks require the suprachiasmatic nuclei (SCN) to synchronize activity, metabolism, and physiology to the external light-dark phase (Dibner et al., 2010). When food is available in the resting phase, the activity pattern of mice changes and food-anticipatory activity (FAA) emerges in addition to the still present nocturnal activity. This FAA persists even in the absence of the SCN, if the food is given every day at the same time (Stephan et al., 1979). Hence, this finding points to a potential involvement of the molecular circadian clock in other brain areas or peripheral tissues. The molecular circadian clock is composed of a set of clock genes that constitute an autoregulatory transcriptional-translational autoregulatory feedback loop (Takahashi, 2017). The positive factors BMAL1 and CLOCK/NPAS2 form heterodimers and bind to E-boxes present in the promoters of target genes such as *Period* (*Per 1-3*), *Cryptochrome* (*Cry1, 2*), and *Rev-erb* (α and β) to activate their transcription. The PER and CRY proteins heterodimerize to enter the nucleus and inhibit the action of the BMAL1-CLOCK/NPAS2 activator complex, whereas REV-ERB represses transcription of the *Bmal1/Clock/Npas2* genes, thereby closing the feedback loop.

Mice lacking *Bmal1* (Storch and Weitz, 2009), *Clock* (Pitts et al., 2003), or *Per1* (Feillet et al., 2006) display normal FAA, whereas in mice that lack *Npas2* (Dudley et al., 2003) or *Cry1/2* (Iijima et al., 2005) FAA is reduced. In mice with a deletion in the PAS domain of the PER2 protein, FAA is abolished under 8 h temporal food restriction (RF) and controlling for calorie uptake (Feillet et al., 2006). However, when applying only 4 h temporal RF without controlling calorie uptake, *Per2* knock-out animals displayed normal FAA (Storch and Weitz, 2009; Pendergast et al., 2017). These observations indicate that the circadian clock mechanism itself is probably not causing FAA, but that some components of the clock affect FAA, likely through interaction with other cycling processes such as metabolism. Consistent with this view is the observation that deletion of *Per2* in the liver leads to a lack of FAA, likely by interference with β-hydroxybutyrate (βOHB) production and its subsequent signaling in the brain (Chavan et al., 2016). βOHB, a ketone body, is a small polar molecule which requires active transport through the blood-brain barrier (BBB) (Newman and Verdin, 2014) to serve as a signal to induce motivation to search for food.

Here, we focus on a widely expressed βOHB transporter, the monocarboxylate transporter 1 (MCT1, SLC16a1) (Lengacher et al., 2013; Carneiro and Pellerin, 2015), to investigate its role in RF. Our results show that liver *Mct1* knock-out (KO), but not neuronal or glial *Mct1* KO mice, display reduced FAA, which is paralleled by a reduction of the preprandial βOHB concentration in blood. This result highlights the importance of both liver-derived βOHB and its transport via MCT1 in FA, supporting the notion that liver-derived βOHB is a signaling molecule involved in FA at least under combined caloric and temporal RF.

## Results

### Haploinsufficiency of *Mct1* Is Associated With Reduced FAA

**FIGURE 1:**
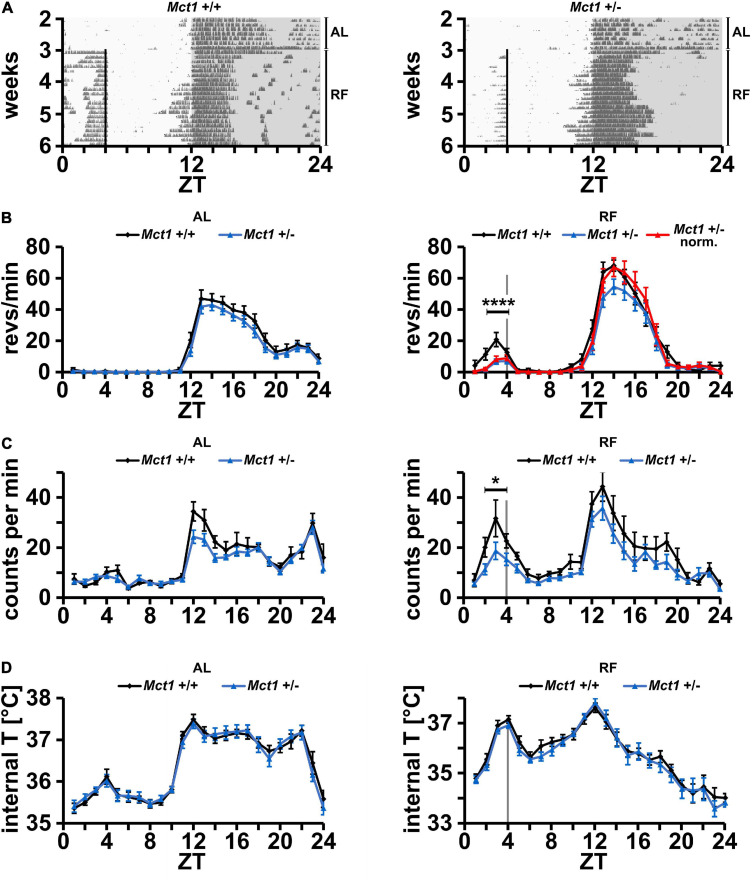
Mice heterozygous for *Mct1* display reduced FAA but have a normal temperature profile. **(A)** Examples of wheel-running actograms of *Mct1*^+/+^ (left panel) and *Mct1*^+/–^ mice (right panel) under *ad libitum* (AL) and daytime-restricted feeding (RF) conditions. The vertical line at ZT4 indicates the time of food access. **(B)** Quantified wheel-running activity plots under AL (left panel) and the last week of RF (right panel) conditions. Food anticipatory activity (FAA) under RF is significantly reduced in *Mct1*^+/–^ (blue) and normalized *Mct1*^+/–^ (red) compared to *Mct1*^+/+^ mice (black). Activity was normalized to dark phase activity of controls (*n* = 13–15, 2-way ANOVA, ****p = 4.89 × 10^–4^, *F* = 13.22). **(C)** Quantified general activity plots under AL (left panel) and RF (right panel) conditions. FAA under RF is significantly reduced in *Mct1*^+/–^ (blue) compared to *Mct1*^+/+^ mice (black) (*n* = 8–9, 2-way ANOVA, *p = 0.01, *F* = 7.02). **(D)** Internal body temperature profiles under AL (left panel) and RF (right panel) conditions. No differences were observed between the genotypes under both AL and RF conditions (*n* = 8–9, 2-way ANOVA, *p* > 0.05). Error bars represent the standard error of mean.

In a previous report, we found that liver-derived ketone bodies play a role in food anticipation (Chavan et al., 2016). We hypothesized that βOHB serves as a signaling molecule that is released from the liver into the bloodstream from where it is transported into the brain to elicit activity to search for food. Since some monocarboxylate transporters (MCTs, SLC16a family) are responsible for the transport of βOHB, notably through the BBB, we aimed to evaluate the importance of the monocarboxylate transporter 1 (*Mct1, Slc16a1*) in food anticipation (FA). Indeed, MCT1 has the broadest selectivity of all MCTs and transports βOHB (Carneiro and Pellerin, 2015). Since homozygous deletion of *Mct1* leads to death at an early embryonic stage (Lengacher et al., 2013), we assessed the activity of haploinsufficient *Mct1* (*Mct1*^ + ⁣/−^) mice. Under *ad libitum* (AL) conditions, control *Mct1*^+/+^ and *Mct1*^ + ⁣/−^ animals both display robust nocturnal wheel-running activity with no activity in the light phase (Figure 1A, AL). Restricting food access from ZT4 to ZT12 (from 4 h after lights on to lights off) with 70% of normal caloric intake elicits a strong activity before ZT4 in control mice (Figure 1A, RF, left panel and Supplementary Figure 1, left panel), whereas for the *Mct1*^ + ⁣/−^ animals this activity is strongly reduced (Figure 1A, RF, right panel and Supplementary Figure 1, right panel). Quantification of wheel-running activity revealed no difference in locomotion between the two genotypes under AL conditions (Figure 1B, left panel). Under RF conditions, activity was reduced in *Mct1*^ + ⁣/−^ animals before ZT4 in anticipation of food, and between ZT12 and ZT24 (dark phase) their activity was also slightly lower compared to controls (Figure 1B, right panel). Normalization of the activity in the dark phase revealed that FAA before ZT4 was reduced compared to controls (Figure 1B, right panel, red), indicating that *Mct1* may play a role in the regulation of FAA. In addition to the difference in total wheel revolutions before feeding time (Supplementary Figure 2A and Supplementary Table 1), the mice from the haploinsufficient group appeared to have a dyssynchronous onset of activity before food availability. This is apparent when the pooled data are viewed at 10 min resolution, showing a less steep FAA activity onset compared to that of the control group (Supplementary Figure 3).

To corroborate this result, we also tested general activity of mice via an implanted wireless biochip, which allowed simultaneous measurements of body temperature (see section “Materials and Methods”). Consistent with the activity in the running-wheel, we observed that control and *Mct1*^ + ⁣/−^ animals did not significantly differ in their activity under AL conditions (Figure 1C, left panel). Under RF conditions, a reduction of FAA was seen in *Mct1*^ + ⁣/−^ mice compared to controls (Figure 1C, right panel), similar to the observation in the running-wheel activity assessment (Figure 1B, right panel). Interestingly, however, the genotypes did not differ in their body temperature profile over time under both AL and RF conditions (Figure 1D). This indicates that *Mct1* plays a role in FAA, but does not influence the anticipatory temperature increase in response to RF.

To study *Mct1*’s role in FAA in a tissue-specific manner, we used mice with a conditional *Mct1* allele (Zhang et al., 2020; Boucanova et al., 2021; Supplementary Figure 4A) and crossed them with tissue-specific *Cre* lines. Since homozygous *Mct1* total body deletion were not viable, we explored if knockout of both *Mct1* alleles from the liver, neurons or glia was compatible with life. To our surprise, our matings of *Mct1*^*fl/fl*^ mice with different *Cre*-expressing lines showed that homozygous liver (*Alb1*-*Cre*^+^ *Mct1*^*fl*/*fl*^), neuronal (*Nes*-*Cre*^+^ *Mct1*^*fl*/*fl*^) and glial (*Gfap*-*Cre*^+^ *Mct1*^*fl*/*fl*^) knockout mice were all viable (Supplementary Figure 4). Whereas the liver *Cre* line showed high specificity of recombination, consistent with our previous observations (Chavan et al., 2016), we were able to detect deleted alleles outside of the central nervous system (CNS) in case of the *Nestin* and *Gfap* knock-outs (Supplementary Figure 4B), consistent with reports of germline recombination and developmental effects of CNS *Cre* lines (Luo et al., 2020).

### Deletion of *Mct1* in *Nestin*-Positive Cells Does Not Affect FAA

**FIGURE 2:**
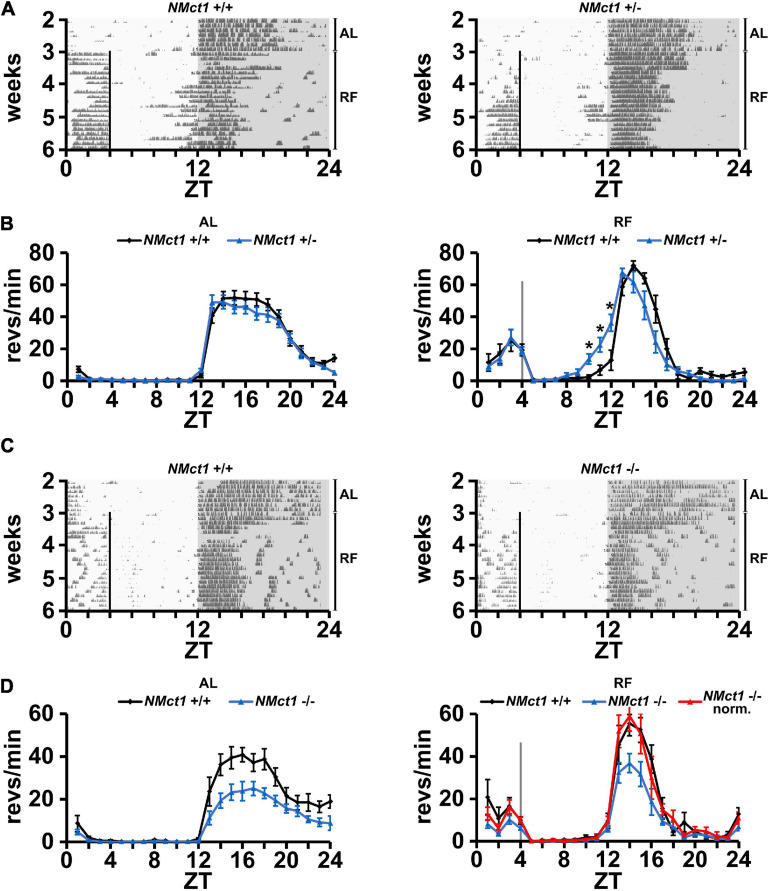
Mice lacking *Mct1* in neurons display normal FAA. **(A)** Examples of wheel-running actograms of neuronal *Mct1* control animals (*NMct1*^+/+^, left panel) and heterozygous neuronal *Mct1* knock-out animals (*NMct1*^+/–^, right panel) under *ad libitum* (AL) and day time restricted feeding (RF) conditions. The vertical line at ZT4 indicates the time of food access. **(B)** Quantified wheel-running activity plots under AL (left panel) and the last week of RF (right panel) conditions. Food anticipatory activity (FAA) under RF is normal in *NMct1*^+/–^ (blue) compared to *NMct1*^+/+^ mice (black) (*n* = 8–12, 2-way ANOVA, *p* > 0.05). Interestingly, onset of activity in *NMct1*^+/–^ animals is significantly earlier compared to *NMct1*^+/+^ controls (*p = 0.02 at individual time-points ZT 10-1, single-point 2-tailed Student’s *t*-test due to non-predefined interval). **(C)** Examples of wheel-running actograms of neuronal *Mct1* control animals (*NMct1*^+/+^, left panel) and homozygous neuronal *Mct1* knock-out animals (*NMct1*^–/–^, right panel) under *ad libitum* (AL) and day time restricted feeding (RF) conditions. The vertical line at ZT4 indicates time of food access. **(D)** Quantified wheel-running activity plots under AL (left panel) and RF (right panel) conditions. Food anticipatory activity (FAA) under RF is normal in *NMct1*^–/–^ (blue) and normalized *NMct1*^–/–^ (red) compared to *NMct1*^+/+^ mice (black) (*n* = 6, 2-way ANOVA, *p* > 0.05). Error bars represent the standard error of mean.

In order to evaluate neuronal effects of *Mct1* expression, we deleted *Mct1* in neurons of floxed *Mct1* mice (Zhang et al., 2020; Boucanova et al., 2021; Supplementary Figure 2A) by crossing them with a *Nestin-Cre* mouse strain (Tronche et al., 1999). Importantly, the strategy of deleting *Mct1* from *Nestin*-positive cells encompasses deletion from MCT1-rich tanycytes, which line the walls of the third ventricle, next to orexigenic and anorexigenic hypothalamic neurons (Lee et al., 2012). For means of simplification, we will refer to this mouse line as neuronal *Mct1* (*NMct1*). Control animals (*NMct1*^+/+^) and neuron-deleted *Mct1* (*NMct1*^+/–^ or *NMct1*^–/–^, Supplementary Figure 4) mice were subjected to wheel-running activity assessment under AL and RF conditions. Under AL conditions, activity of *NMct1*^+/–^ and control animals was comparable (Figures 2A,B, left panel), whereas *NMct1*^–/–^ mice showed reduced activity in the dark phase compared to controls (Figures 2C,D, left panel). Under RF conditions, FAA was comparable between *NMct1*^+/–^ and control mice, but the onset of activity before the dark phase was advanced (Figure 2B, right panel). The *NMct1*^–/–^ animals showed normal FAA and reduced activity in the dark phase, but not an advanced onset of activity at ZT12 (Figure 2D, right panel).

Taken together, our results indicate that deletion of *Mct1* in *Nestin*-positive cells does not affect FAA.

### Deletion of *Mct1* in Astroglia Does Not Affect FAA

**FIGURE 3:**
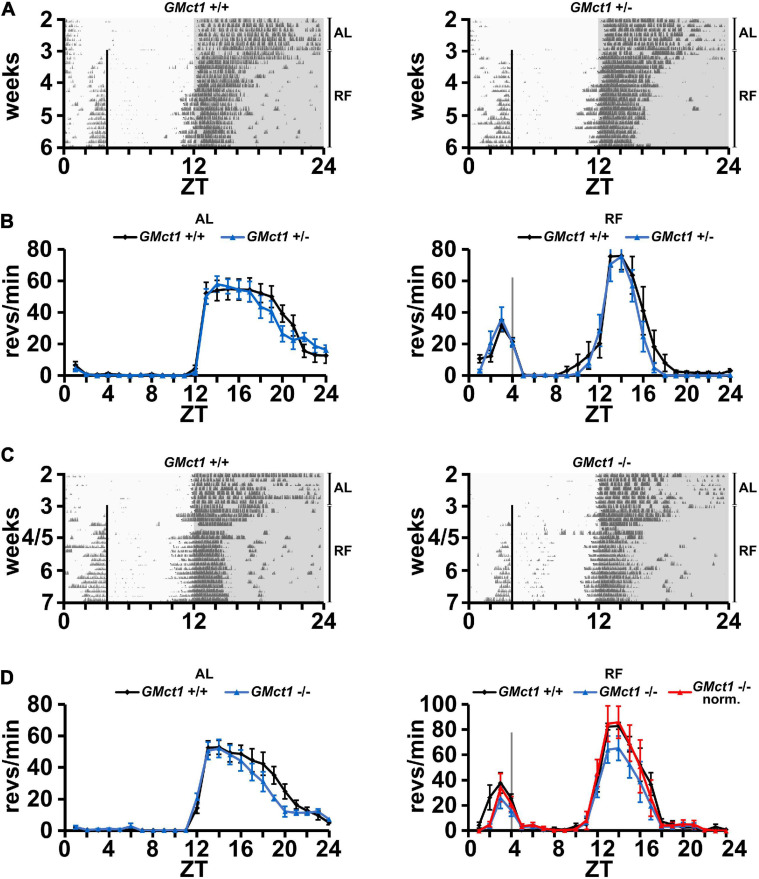
Mice lacking *Mct1* in astroglia display normal FAA. **(A)** Examples of wheel-running actograms of glial *Mct1* control animals (*GMct1*^+/+^, left panel) and heterozygous glial *Mct1* knock-out animals (*GMct1*^+/–^, right panel) under *ad libitum* (AL) and the last week of daytime-restricted feeding (RF) conditions. The vertical line at ZT4 indicates the time of food access. **(B)** Quantified wheel-running activity plots under AL (left panel) and RF (right panel) conditions. Food anticipatory activity (FAA) under RF is normal in *GMct1*^+/–^ (blue) compared to *GMct1*^+/+^ mice (black) (*n* = 6, 2-way ANOVA, *p* > 0.05). **(C)** Examples of wheel-running actograms of glial *Mct1* control animals (*GMct1*^+/+^, left panel) and homozygous glial *Mct1* knock-out animals (*GMct1*^–/–^, right panel) under *ad libitum* (AL) and day time restricted feeding (RF) conditions. The vertical line at ZT4 indicates the time of food access. **(D)** Quantified wheel-running activity plots under AL (left panel) and RF (right panel) conditions. Food anticipatory activity (FAA) under RF is normal in *GMct1*^–/–^ (blue) and normalized *GMct1*^–/–^ (red) compared to *GMct1*^+/+^ mice (black) (*n* = 4–7, 2-way ANOVA, *p* > 0.05). Error bars represent the standard error of mean.

Since *Mct1* is largely expressed by glial cells of the CNS, which includes astrocytes (Pierre and Pellerin, 2005), we deleted *Mct1* in these cells by crossing floxed *Mct1* mice (Zhang et al., 2020; Boucanova et al., 2021) with a *Gfap-Cre* mouse strain (Jackson Lab, stock no. 004600; Zhuo et al., 2001; Luo et al., 2020). The resulting control animals (*GMct1*^+/+^) and glia-deleted *Mct1* mice (*GMct1*^+/–^ and *GMct1*^–/–^, Supplementary Figure 4) were subjected to wheel-running activity assessment under AL and RF conditions. Under AL conditions, activity of *GMct1*^+/–^ and control animals was comparable (Figures 3A,B, left panel), as was the activity of *GMct1*^–/–^ mice and controls (Figures 3C,D, left panel). Under RF conditions, FAA was comparable between *GMct1*^+/–^ and control mice (Figure 3B, right panel). The *GMct1*^–/–^ animals showed overall reduced activity and normalization of the data to dark-phase activity confirmed that FAA was normal in these mice (Figure 3D, right panel).

These data suggest that lack of *Mct1* in glial cells does not compromise FAA.

### Deletion of *Mct1* in Hepatocytes Diminishes FAA

**FIGURE 4:**
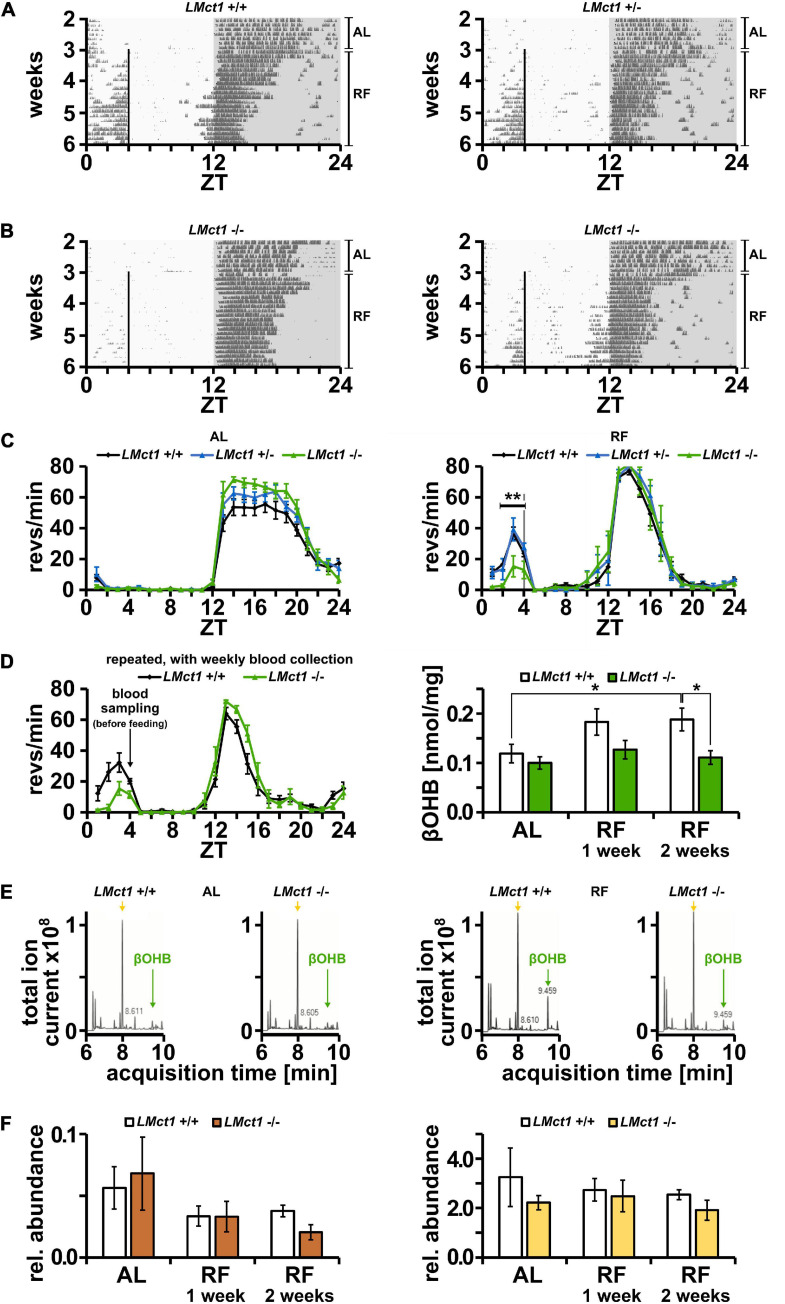
Mice lacking *Mct1* in liver display reduced FAA and show reduced βOHB levels. **(A)** Examples of wheel-running actograms of liver *Mct1* control animals (*LMct1*^+/+^, left panel) and heterozygous liver *Mct1* knock-out animals (*LMct1*^+/–^, right panel) under *ad libitum* (AL) and the last week of daytime-restricted feeding (RF) conditions. The vertical line at ZT4 indicates time of food access. **(B)** Two examples of wheel-running actograms of homozygous liver *Mct1* knock-out animals (*LMct1*^–/–^) under *ad libitum* (AL) and day time restricted feeding (RF) conditions. The vertical line at ZT4 indicates time of food access. Note the reduced activity before feeding time. **(C)** Quantified wheel-running activity plots under AL (left panel) and RF (right panel) conditions. Food anticipatory activity (FAA) under RF is normal in *LMct1*^+/–^ (blue) compared to *LMct1*^+/+^ mice (black), but *LMct1*^–/–^ (green) mice show significantly reduced FAA (*n* = 4–15, 2-way ANOVA, **p = 1.88 × 10^–3^, *F* = 10.71). **(D)** Quantified wheel-running activity plots (left panel) of an additional experiment of *LMct1*^+/+^ (black) and *LMct1*^–/–^ (green) animals under RF conditions (*n* = 4–8), from which blood was drawn at ZT4 just before feeding. Their blood βOHB levels under AL and RF conditions (right panel) show that βOHB levels significantly increase under RF conditions in control mice (*LMct1*^+/+^; *p* = 0.04), whereas these levels stay low in *LMct1*^–/–^ animals and do not significantly increase (*n* = 4–8, 2-tailed *t*-test, *p < 0.05). **(E)** Examples of representative βOHB measurements using mass spectrometry under AL (left panel) and RF (right panel) conditions. Green arrows at 9.46 min indicate the peak of βOHB and yellow arrows at 8.00 min indicate the peak corresponding to lactate. Pyruvate, with a retention time of 8.39 min, is not visible in the TIC (amount too low). **(F)** In contrast to βOHB, relative levels of pyruvate (left panel) or lactate (right panel), expressed as AUC of these molecules compared to the AUC of the internal standard homoserine, do not increase under RF in the control or *LMct1*^–/–^ animals. Error bars represent the standard error of mean.

*Mct1* is not only expressed in the brain, but in many tissues, including the liver (Lengacher et al., 2013). Since βOHB is produced in the liver and needs to be transported into the bloodstream, potentially involving MCT1, we tested the role of liver *Mct1* in FAA. For this purpose, we deleted *Mct1* in hepatocytes of floxed *Mct1* mice (Zhang et al., 2020; Boucanova et al., 2021) by crossing them with an albumin-*Cre* mouse strain (Kellendonk et al., 2000). The resulting control animals (*LMct1*^+/+^) and hepatocyte-deleted *Mct1* mice (*LMct1*^+/–^ or *LMct1*^–/–^, Supplementary Figure 4) were subjected to wheel-running activity assessment under AL and RF conditions. Under AL conditions, activity of *LMct1*^+/–^, *LMct1*^–/–^ and control animals was comparable (Figures 4A–C, left panel). Under RF conditions, FAA was comparable between *LMct1*^+/–^ and control mice, but significantly reduced in *LMct1*^–/–^ mice (Figures 4B,C and Supplementary Figure 5). This reduction of FAA was comparable to the one observed in haploinsufficient *Mct1* animals (Figure 1).

In order to test the consequence of lack of *Mct1* in the liver, we measured βOHB levels in the blood, collected weekly at ZT4 just before feeding. The activity profile of *LMct1*^–/–^ and control animals during this feeding with weekly blood sampling confirmed our initial observations (Figure 4C, right panel) that *LMct1*^–/–^ mice display reduced FAA (Figure 4D, left panel). Under AL conditions, both genotypes show comparable amounts of βOHB in the blood (Figure 4D, right panel). In contrast, under RF conditions only the control animals increase their βOHB levels in the blood, whereas in *LMct1*^–/–^ mice βOHB amounts remain at a similar level as under AL conditions (Figure 4D, right panel). Examples of mass spectrometry analysis of βOHB in both genotypes under AL and RF conditions are shown in Figure 4E (green arrow = βOHB, yellow arrow = lactate). Pyruvate and lactate, which are also transported by MCT1, do not increase under RF (Figure 4F, left panel = pyruvate, right panel = lactate).

These results indicate that liver *Mct1* plays an important role in the regulation of βOHB levels in the blood. Reduced βOHB amounts may then lower FAA as exemplified in *LMct1*^–/–^ mice. This interpretation is consistent with our previous studies, which showed that timed release of βOHB with implanted programmable mini-pumps rescues FAA in liver *Per2* KO mice (Chavan et al., 2016).

### *Mct1* Expression Adapts to Feeding Time

**FIGURE 5:**
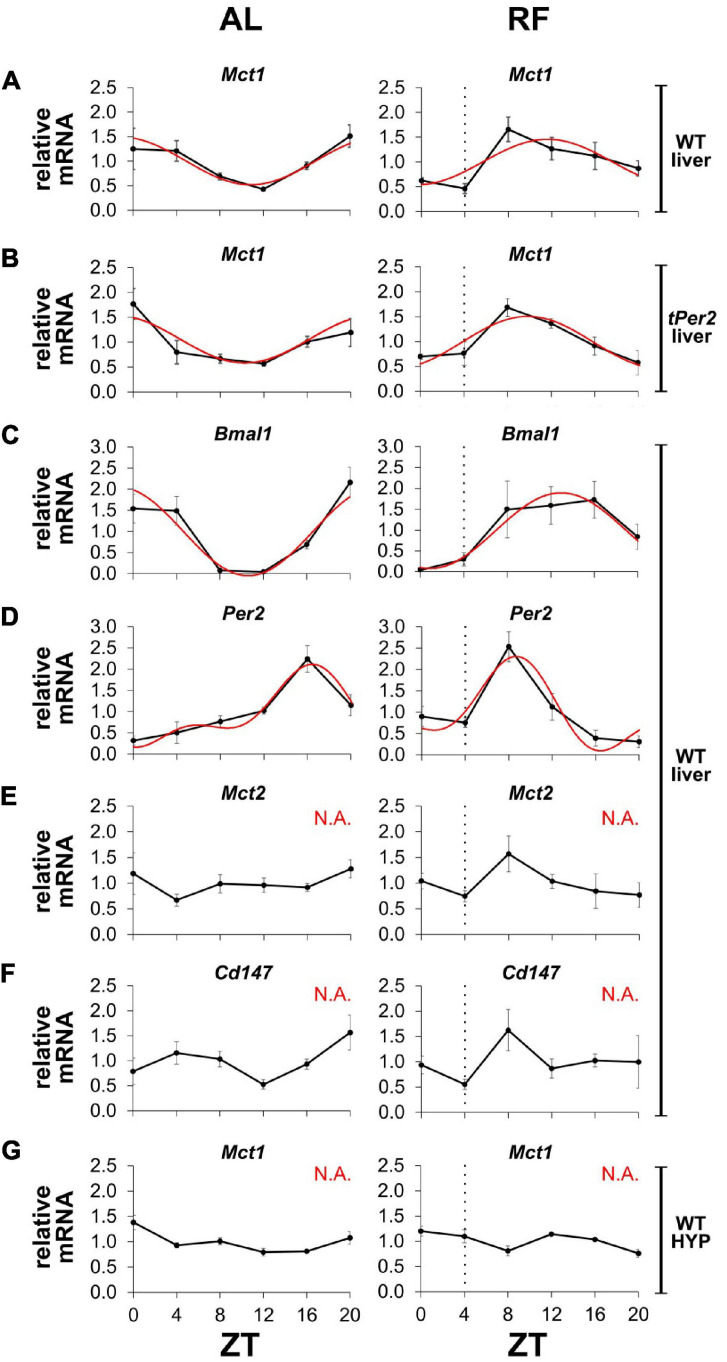
*Mct1* expression adapts to feeding time. mRNA expression under *ad libitum* (AL, left panels) and restricted feeding (RF, right panels) conditions. The black hatched vertical line indicates feeding time. **(A)** *Mct1* expression in liver of wild-type (WT) mice. **(B)** *Mct1* expression in liver of total *Per2* knock-out (*tPer2*) animals. **(C)** *Bmal1*, **(D)** *Per2*, **(E)** *Mct2*, and **(F)** *Cd147*, expression in liver of WT mice. **(G)** *Mct1* expression in the hypothalamus (HYP) of WT animals. Tissue at ZT4 was harvested just before feeding. Red lines represent fitted curves, calculated with CircWave 1.4 (R. Hut). Based on the fits, *Mct1* in WT animals peaks at ZT 22.8, whereas in total *Per2* KO animals it peaks at 22.3. *Bmal1* peaks at 22.6 and 13.0, respectively, and *Per2* at 16.4 and 8.7. Under RF, the expression of *Mct1* in WT animals is advanced 11.11 h (AL: *p* = 4.2 × 10^–3^, *R*^2^ = 0.517; RF: *p* = 1.2 × 10^–2^, *R*^2^ = 0.443), while in total *Per2* KO animals the advance is 12.49 h (AL: *p* = 4.7 × 10^–3^, *R*^2^ = 0.510; RF: *p* = 1.1 × 10^–3^, *R*^2^ = 0.598). *Bmal1* is advanced 9.65 h (AL: *p* = 4.9 × 10^–5^, *R*^2^ = 0.734; RF: *p* = 3.6 × 10^–3^, *R*^2^ = 0.527), whereas *Per2* advances 7.73 h (AL: *p* = 2.2 × 10^–4^, *R*^2^ = 0.794; RF: *p* = 1.6 × 10^–3^, *R*^2^ = 0.717). “N.A.” indicates that a fit was not possible (alpha < 0.05). Error bars represent the standard error of mean.

Since MCT1 is important for the release of βOHB from the liver into the bloodstream, we wanted to investigate whether expression of *Mct1* was influenced by feeding time. We submitted mice to AL or RF conditions and collected liver tissue around the clock in order to determine expression of several genes using qPCR (Figure 5). We observed that temporal expression of *Mct1* in the liver, which peaked at ZT20 under AL conditions, adapted to RF with maximal expression at ZT8, covering the time span from restricted food access at ZT4 to ZT8 (Figure 5A). Interestingly, this change in *Mct1* expression in the liver was not affected in mice lacking the clock gene *Per2*, indicating that *Mct1* adaptation to RF was independent of *Per2* (Figure 5B). It is possible that the adaptation was directly influenced by metabolic conditions of RF, during which tissue was isolated. The clock genes *Bmal1* and *Per2* both changed their expression in the liver in response to RF. Of note is that also *Mct2* adapted to RF conditions (Figure 5E), as did the chaperone partner of *Mct1*, basigin (*Cd147*), which is required for proper plasma membrane insertion of MCT1 (Figure 5F). Remarkably, *Mct1* expression in the hypothalamus was unaffected by RF and comparable to AL conditions (Figure 5G). The data was further analyzed with CircWave 1.4 by applying forward linear harmonic regression to the measurements, which resulted in sinusoidal fits of gene expression over time and an estimation of the quality of the fit (Figure 5, red curves). Interestingly, whereas a robust fit was possible in the case of liver *Mct1*, but not hypothalamic *Mct1* (Figure 5G), we could not fit a curve on liver *Mct2* or liver *Cd147* expression data (Figures 5E,F).

Taken together, our results indicate that expression of *Mct1* and *Cd147* in the liver but not the hypothalamus adapts to RF. Although expression of the clock genes *Bmal1* and *Per2* adapt to RF in the liver as well, *Per2* appears not to be responsible for the adaptation of *Mct1* to RF.

## Discussion

βOHB has long been known as a source of energy circulating in the blood, but only recently it has been recognized as a signaling molecule (Newman and Verdin, 2014). βOHB can be produced in the liver from where it is released into the bloodstream by monocarboxylate transporters (MCTs). Once in the vascular system, βOHB can be imported by extrahepatic organs via MCTs or bind to cell-surface G-protein coupled receptors (GPCRs), such as hydroxycarboxylic acid receptor 2 (HCAR2/GPR109) and free fatty acid receptor 3 (FFAR3/GPR41) to reduce lipolysis, reduce sympathetic tone and lower metabolic rate (Kimura et al., 2011). In the target organ, βOHB can then be used either as an energy source in the TCA cycle and/or it can bind and inhibit histone deacetylases (HDACs) (reviewed in Newman and Verdin, 2014). This leads to hyperacetylation of proteins (e.g. histones, p53), which is generally associated with gene expression.

In this study we were interested in the question how βOHB from the liver can act as a signal on the brain to produce FAA in response to RF. In particular, we focused on one of the βOHB membrane transporters, MCT1. In order to gain insight whether this membrane transporter is important for βOHB export from the liver, import to neurons or import to astrocytes to elicit FAA, we deleted *Mct1* either in the entire animal or specifically in hepatocytes, neurons or glia.

Total deletion of *Mct1* was described to be lethal, but whole-body hemizygous mice were resistant to diet-induced obesity and showed metabolic perturbations (Lengacher et al., 2013). We exposed these animals to caloric and temporal RF and compared them to the corresponding control animals (Figure 1). The *Mct1*^+/–^ animals displayed a significantly reduced response to RF at ZT4 as manifested by reduced FAA. This indicated that *Mct1* most likely plays an important role in the regulation of FAA at the level of locomotor activity (Figures 1A–C), but not at the level of temperature regulation (Figure 1D). This phenotype is reminiscent of the behavior of *Per2* total knock-out and *Per2* liver knock-out animals, which display reduced βOHB levels in response to caloric/temporal RF (Chavan et al., 2016). However, in contrast to liver *Per2* knock-out mice, *Mct1* haploinsufficient animals show unaltered internal body temperature profiles under RF (Figure 1D). Since *Per2* regulates βOHB synthesis, but *Mct1* acts downstream of βOHB synthesis and is involved in its export, one may speculate that liver metabolism of ketone bodies is involved in body temperature regulation in response to RF. This may also involve mechanisms regulated by the CNS (Gooley et al., 2006; Bartfai and Conti, 2012) that we did not investigate here and is subject of further research.

Since βOHB is released from the liver to reach the brain via the bloodstream, we tested whether *Mct1* in the brain is important for receiving the βOHB signal to elicit FAA. Therefore, we deleted *Mct1* in *Nestin-* and *Gfap-*positive cells, respectively, and tested the resulting *NMct1* (Figure 2) and *GMct1* (Figure 3) knock-out animals for FAA in response to RF. Both, *NMct1* and *GMct1* animals displayed normal FAA, although *NMct1* homozygous KOs showed reduced activity in the dark phase in both AL and RF conditions (Figures 2, 3). For the *NMct1* mice, normal FAA was expected, because *Mct1* is mainly expressed in astrocytes and not in neurons (Pierre and Pellerin, 2005). In contrast, *Mct1* is well expressed in astrocytes and therefore the normal response of *GMct1* mice to RF was somewhat unexpected. This may be due to the fact that βOHB can bind to GPCRs, such as HCAR2/GPR109 first described as a nicotinic acid receptor (Tunaru et al., 2003; Taggart et al., 2005; Offermanns et al., 2011), and FFAR3/GPR41 (Kimura et al., 2011), and therefore may modulate feeding behavior through these receptors. The normal response of *NMct1* and *GMct1* mice to RF may also be the consequence of developmental adaptation, and/or compensation by another transporter of the *Mct* family. Taken together, our observations suggest that *Mct1* in neurons or glia may not be crucial for βOHB signaling to elicit FAA, however, we cannot exclude that absence of *Mct1* in both cell types at the same time may affect FAA. In order to show the temporal dynamics of FAA establishment, which could be altered in the *NMct1* or *GMct1* animals, we quantified onset also during the first week of RF (Supplementary Figure 6). Both genotypes showed FAA similar to their controls, indicating that establishment of FAA was not significantly different to control animals.

The hemizygous *Mct1* and homozygous *Per2* liver knock-out animals both show reduced FAA in response to RF (Figure 1; Chavan et al., 2016). Therefore, we deleted *Mct1* specifically in the liver (*LMct1*) in order to evaluate liver *Mct1* function for the generation of FAA in response to RF. We observed that *LMct1*^–/–^ but not *LMct1*^+/–^ animals displayed reduced, but not absent FAA (Figures 4A–C), comparable to the whole body hemizygous *Mct1* mice (Figures 1A–C). This indicates that *Mct1* in the liver is involved but is not sufficient to elicit FAA. Since *Mct1* is expressed throughout the body (Pierre and Pellerin, 2005), other organs are most likely involved in the establishment of FAA in response to RF and hence MCT1 probably coordinates at least in part βOHB signaling.

That coordination of βOHB signaling is important was suggested by the adaptation of *Mct1* and *Cd147* expression to RF (Figures 5A,F). Although the clock genes *Bmal1* and *Per2* adapt to RF as well (Figures 5C,D), it appears that the clock may not be directly responsible for the shift of *Mct1* expression, because *Mct1* adapted to RF in the absence of the *Per2* gene (Figure 5B). This is consistent with transcriptional landscape analysis of clock genes, which showed that none of the clock genes bound to the promoter of *Mct1* in liver tissue over 24 h (Koike et al., 2012). The nuclear receptor PPARα is known to have a crucial role in adaptation to fasting (Kersten et al., 1999), and therefore has been proposed to regulate *Mct1* expression based on cell culture experiments (Konig et al., 2008; Konig et al., 2010). Although fasting of mice did increase *Mct1* expression in the liver, a similar increase was observed in mice lacking PPARα, suggesting no role of PPARα in the regulation of *Mct1* (Schutkowski et al., 2014). This is consistent with our observation that *Per2*, whose protein binds and modulates PPARα activity (Schmutz et al., 2010; Chappuis et al., 2013), played no role in adaptation of *Mct1* expression to RF (Figure 5B). Hence, other metabolic and/or hormonal feeding signals are involved in this adaptation process.

βOHB appeared to be released in response to RF in wild-type control animals, but not in mice lacking *Mct1* in the liver (Figures 4D,E). This is paralleled by lack of FAA, but absence of *Mct1* in neurons or glia resulted in normal FAA (Figures 2, 3). Hence, *Mct1* is important for the release of βOHB, but the receiving end of βOHB signaling appeared not to be affected by lack of *Mct1*. Therefore, βOHB may signal normally and stimulate feeding due to increasing agouti related peptide (*Agrp*) and neuropeptide Y (*Npy*) expression in the hypothalamus (Park et al., 2011). Hence, for further investigations of βOHB signaling, hypothalamic structures such as the arcuate nucleus will be relevant. Of note is that we cannot distinguish between clock-controlled and metabolically stimulated locomotor activity. Therefore, we can only state that *Mct1* and βOHB are important for the expression of FAA, but we do not know whether they are regulating circadian timing of FAA.

In this study we provide evidence that βOHB signaling is affected by lack of *Mct1* in the liver, but not by lack of it in neuronal or glial cells. Lack of *Mct1* in the liver leads to a reduced release of βOHB in response to combined caloric/temporal RF, which is paralleled by reduced FAA. Hence, we postulate that *Mct1* regulates FAA by controlling the release of βOHB from the liver into the bloodstream under caloric and temporal RF.

## Materials and Methods

### Animals

Generation of *Mct1*^+/–^ mice (Slc16a1^TM 2.1*Lupel*^) has been described in detail elsewhere (Lengacher et al., 2013). Briefly, 640 bp of the *Mct1* gene with exon 1 and part of intron 1 were replaced by *LacZ* fused with a neomycin resistance sequence, in frame with the *Mct1* promoter, to create *Mct1*^+/–^ mice. *Mct1* floxed (*Mct1*^*fl*/*fl*^) mice (Slc16a1^TM 1.1*Lupel*^) were generated by flanking exon 5 with *LoxP* sites, allowing for *Cre* deletion (Supplementary Figure 2; Zhang et al., 2020; Boucanova et al., 2021). The albumin-driven *Cre* line [Tg(Alb1-cre)7Gsc/Ibcm: Tg alfpCre], *LCre*, was obtained from the European Mouse Mutant Archive (EM: 00603) (Kellendonk et al., 2000), the nestin- [Bclaf1 × Tg Nes-cre C57BL/6: Tg(Nes-cre)1Kln], *NCre*, was received from FMP Leibnitz-Institute for Molecular Pharmacology (EMMA EM:04561) (Tronche et al., 1999), and the glial fibrillary acidic protein-driven *Cre* line [FVB-Tg(GFAP-cre)25Mes/J], *GCre*, was from Jackson Lab (stock no. 004600) (Zhuo et al., 2001; Luo et al., 2020). All mice were backcrossed to the C57BL/6 strain for at least 10 generations. For means of simplification *LMct1*, *NMct1* and *GMct1* are used to denote liver, neuronal and astroglial *Mct1* KO mice, respectively. Male and female animals of 2–4 months of age and a body weight of 25–30 g were used in this study in all experiments and no differences between the sexes were observed.

Housing as well as experimental procedures were performed in accordance with the guidelines of the Schweizer Tierschutzgesetz and the Declaration of Helsinki. The state veterinarian of the Canton of Fribourg and Bundesamt für Umwelt BAFU approved the protocols. The study was carried out in compliance with the ARRIVE guidelines.

### The Restricted Feeding Protocol and Recordings of Activity and Temperature

The restricted feeding protocol, together with the recordings, has been described in detail elsewhere (Martini et al., 2019). Briefly, mice were single-caged and the activity or temperature was recorded for 3 weeks under free access to food, which we denote as *ad libitum* (AL), and then for 3 weeks under daytime restriction (RF), with mice receiving 80% of their daily intake (measured during AL) in the first week and 70% in the following 2 weeks. Mice were fed at ZT4 (4 h after lights on) and food was removed at ZT12 (just before lights off). When entrained to RF, mice ate their portion of food during the first 3–4 h. During experiments, the temperature was maintained at 25 ± 2°C. Activity was recorded based on revolutions of a running-wheel, which was mounted in the cage, and activity patterns were acquired and analyzed using the Actimetrics ClockLab software, Version 3.0 acquisition and 6.0.54 analysis. Internal body temperature was recorded using wireless implantable chips G2 E-Mitter from Starr Life Sciences and analyzed using the VitalView software Version 5.0. Activity and temperature measurements of the last week of AL and RF were analyzed. Due to a transient drop of the temperature in our facility during the feeding of *GMct1*^–/–^ mice, the RF protocol was extended for 1 week and the last week was analyzed.

Preprandial changes in activity and temperature profiles were compared using a 2-way ANOVA, with independent variables of ZT and genotype. Time-points ZT 2, 3, and 4 were used for analysis at 1 h resolution, whereas time-points from and including ZT 2 to and including ZT 4 were used for analysis at 10 min resolution. The *p*-value and *F*-value of the effect of genotype on wheel revolutions is noted in corresponding figure legends and in Supplementary Table 2. RStudio Version 1.1.456 was used for the analysis.

### Quantification of βOHB, Pyruvate, and Lactate in Whole Blood

Whole blood was collected at ZT4 (feeding time) from the lateral tail vein, before food was given to the animals. Blood was immediately weighed on an analytical scale and flash frozen in liquid nitrogen, followed by analysis of βOHB content. Briefly, blood was mixed with extraction buffer (homoserine as the internal standard in 80% ethanol) and centrifuged. A proportion of the sample was dried with evaporation, resuspended in acetonitrile: BSTFA [N,O-sis(trimethylsilyl)trifluoroacetamide; ratio 1 : 1] and incubated at 75°C for 30 min. GC-MS was carried out using a GC-Q/TOF 7250 (Agilent) equipped with a 30 m HP5-MS ultra-inert (30 m × 250 μm, 0.25 μm) column. Injection volume was 1 μl at 250°C and a carrier gas flow of 1 ml/min helium was used, with a split ratio of 100 : 1. The initial oven temperature of 50°C was maintained for 2 min and then raised to 300°C at 10°C/min. βOHB in samples was identified by comparison of the retention time with the authentic standard and by the presence of four qualifier fragments (m/z 117, 147, 191 and 233). The EI-MS also matches with the reference spectrum in the NIST (National Institute of Standards and Technology) Library (Supplementary Figures 7A–D). Quantification was based on a 6-point calibration curve done in parallel with sample analysis (Supplementary Figure 7E). In the same samples, pyruvate and lactate were also identified based on the reference spectrum and the fragments and retention time of their authentic standard. Relative quantification was performed by comparing the area under the curve (AUC) of pyruvate or lactate with the AUC of the internal standard homoserine.

### Gene Expression Analysis

Hypothalamic and liver tissue for gene expression analysis was collected at 4 h intervals. In the case of liver, blood was flushed from the organ prior to isolation by injecting 5–10 ml of saline into the spleen of a decapitated animal. RNA was isolated using the Macherey-Nagel NucleoSpin RNA Plus kit, according to the manufacturer’s instructions. The homogenization step was performed using a motorized pestle. Isolated RNA was reverse transcribed using the Superscript II Reverse Transcriptase (Sigma-Aldrich). Relative mRNA was quantified by real-time PCR on a RotorGene-6000 (Labgene). For the list of primers see Table 1.

**TABLE 1:** Primers.

**qPCR**
***18S***
FW: 5′-CCG GCG GCT TGG TGA CTC TA-3′
RV: 5′-GGC AGA CGT TCG AAT GGG TCG T-3′
PR: 5′-FAM-CCT CGG GCC GAT CGC ACG CC-BHQ-1-3′
***Mct1***
FW: 5′-AAT GCT GCC CTG TCC TCC TA-3′
RV: 5′-CCC AGT ACG TGT ATT TGT AGT CTC CAT-3′
***Mct2***
FW: 5′-CAG CAA CAG CGT GAT AGA GCT-3′
RV: 5′-TGG TTG CAG GTT GAA TGC TAA-3′
***Mct4***
FW: 5′-CAG CTT TGC CAT GTT CTT CA-3′
RV: 5′-AGC CAT GAG CAC CTC AAA CT-3′
***Cd147***
FW: 5′-GCT GGC CTT CAC GCT CT-3′
RV: 5′-CTG CGA CAG TGG TGC CTT-3′
***Tspo***
FW: 5′-GGT CAG CTG GCT CTG AAC TG-3′
RV: 5′-CAG TCG CCA CCC CAC TGA CA-3′
TM: 5′-TGC CCG GCA GAT GGG CTG GGC-3′
***Per2***
FW: 5′-TCC ACA GCT ACA CCA CCC CTT A-3′
RV: 5′-TTT CTC CTC CAT GCA CTC CTG A-3′
PR: 5′-FAM-CCG CTG CAC ACA CTC CAG GGC G-BHQ-1-3′
***Bmall***
FW: 5′-CCAAGAAAGTATGGACACAGACAAA-3′
RV: 5′-GCATTCTTGATCCTTCCTTGGT-3′
PR: 5′-FAM-TGACCCTCATGGAAGGTTAGAATATGCAGAA-TAMRA-3′
****Genotyping****
***Forward blue***
FW: 5′-TCT CCC TGT AGC ACT TGT CAG TTT GTA A- 3′
***Reverse blue***
RV: 5′-GAC TAG AGC TTG CGG AAC CCT T-3′
***Reverse green***
RV: 5′-TCC AAG GAC AGC CAA GCT ACA TAG AG-3′

## Data Availability Statement

The original contributions presented in the study are included in the article/Supplementary Material, further inquiries can be directed to the corresponding author/s.

## Ethics Statement

The animal study was reviewed and approved by the Veterinäramt Kanton Freiburg (license 31905).

## Author Contributions

TM, LP, and UA conceived and designed the experiments. TM, JR, RC, and MS performed the experiments. TM, JR, RC, MS, CN, and UA analyzed the data. LP and UA contributed reagents, materials, and analysis tools. TM and UA wrote the manuscript. All authors contributed to the article and approved the submitted version.

## Conflict of Interest

The authors declare that the research was conducted in the absence of any commercial or financial relationships that could be construed as a potential conflict of interest.

## Supplementary Material

Supplementary Figure 1 Examples of double-plotted wheel-running actograms of *Mct1*^+/+^ (left panel) and *Mct1*^+/–^ mice (right panel) under *ad libitum* (AL) and daytime-restricted feeding (RF) conditions. The vertical line at ZT4 indicates the time of food access.

Supplementary Figure 2 **(A)** Graphical representation of average daily quantified wheel revolutions of *Mct1*^+/+^ and *Mct1*^+/–^ haploinsufficient mice during ZT 2–4 (left panel) and ZT 12–24 (right panel) of their last week of RF. **(B)** Graphical representation of average daily quantified wheel revolutions of *LMct1*^–/–^ mice and their controls during ZT 2–4 (left panel) and ZT 12–24 (right panel) of their last week of RF. The quantification includes the time-points that limit the intervals of quantification (closed intervals). The group comparison of non-normalized and normalized data vs. the control group was performed with the 2-tailed Student’s *t*-test (*n* = 4–15, ^∗^*p* = 0.02 and 0.03, respectively).

Supplementary Figure 3 Quantified wheel-running activity plots under AL **(A)** and the last week of RF **(B)**, as in Figure 2, but with 10 min resolution. FAA was compared between ZT2 and ZT4 with a 2-way ANOVA (*n* = 13–15, ^****^*p* = 7.45 × 10^–14^, *F* = 60.62). The slope of rise in activity before feeding time under RF conditions (yellow line) is significantly flatter in *Mct1*^+/–^ compared to control animals. **(C)** Analysis of the onset of activity by performing linear regression on time points from ZT 1.5 to ZT 2.5 (13 measurements) revealed that the control group had an increase of activity of 26 revolutions of the wheel per 10 min, while the *Mct1*^+/–^ group had an increase of 5, and the *Mct1*^+/–^ normalized 6 revolutions per 10 min, with *R-*squared values of 0.96, 0.89, and 0.89, respectively. Error bars represent the standard error of mean. **(D)** Representative actograms of *Mct1*^+/+^ and *Mct1*^+/–^ mice show that the onset of FAA in the control group is comparable between mice of the group, and it is also robust for each individual mouse, with predictable onsets each day. The mice from the haploinsufficient group show that the FAA onset is different on each day for one single mouse and also within the group.

Supplementary Figure 4 Generation of tissue-specific *Mct1* KO mice. **(A)** Top panel: Diagram of the *Mct1* wild-type allele (*Mct1*^+/+^), conditional KO allele with LoxP sites flanking exon 5 (*Mct1*^*fl/fl*^), and the constitutive KO (*Mct1*^–/–^) allele after Cre recombination. Bottom panel: Genotyping results of the corresponding wild-type, heterozygous and homozygous *Mct1* floxed mice. The band at 280 bp refers to the wild-type allele, and the band at 420 bp indicates the LoxP insertion. **(B)** Genotyping of *LMct1*^–/–^, *NMct1*^–/–^, and *GMct1*^–/–^ mice reveals a deleted allele at 437 bp in the liver tissue of the *Alb1*-*Cre*^+^ *Mct1*^*fl/fl*^ animal and an absence of deleted alleles in the ear biopsy (used for initial genotyping) and the hypothalamus (HYP). In the case of the *Nes*-*Cre* driver, the strongest recombined band is noticed in the HYP, but expectedly a weak band could also be detected in the ear and liver tissue. The *Gfap*-*Cre* driver shows higher specificity, with recombination detected in the HYP and a weak band in the ear biopsy.

Supplementary Figure 5 Examples of double-plotted wheel-running actograms of *LMct1*^+/+^ (left panel) and *LMct1*^–/–^ mice (right panel) under *ad libitum* (AL) and daytime-restricted feeding (RF) conditions. The vertical line at ZT4 indicates the time of food access.

Supplementary Figure 6 Activity profile of first week of RF for **(A)** neuronal, **(B)** glial, and **(C)** liver homozygous *Mct1* KO mice vs. corresponding controls shows that neuronal and glial *Mct1* KO mice show same patterns of adaptation to RF as their corresponding controls, whereas the adaptation of *LMct1*^–/–^ mice is already affected in the first week of RF.

Supplementary Figure 7 Identification of βOHB was done based on **(A)** retention time and **(B)** EI-MS fragments of an authentic standard. βOHB in our sample was eluted **(C)** at the expected retention time of 9.46 min and **(D)** showed fragments identical to those of the authentic standard. **(E)** Quantification was based on a 6-point calibration curve.

Supplementary Table 1 Quantifications of total wheel revolutions between ZT 2 and 4, as well as between ZT 12 and 24 for each group of animals. The quantification includes the time-points that limit the intervals of quantification (closed intervals). The group comparison of non-normalized and normalized data vs. the control group was performed with the 2-tailed Student’s *t*-test (*n* = 4–15).

Supplementary Table 2 Results of statistical analysis of comparison of FAA ZT2-4 using 2-way ANOVA.
