# Supplementary figures and images for: The Hepatic Monocarboxylate Transporter 1 (MCT1) Contributes to the Regulation of Food Anticipation in Mice
Source: Front Physiol. 2021 Apr 14;12:665476. doi: 10.3389/fphys.2021.665476 (PMC8079775; doi:10.3389/fphys.2021.665476)

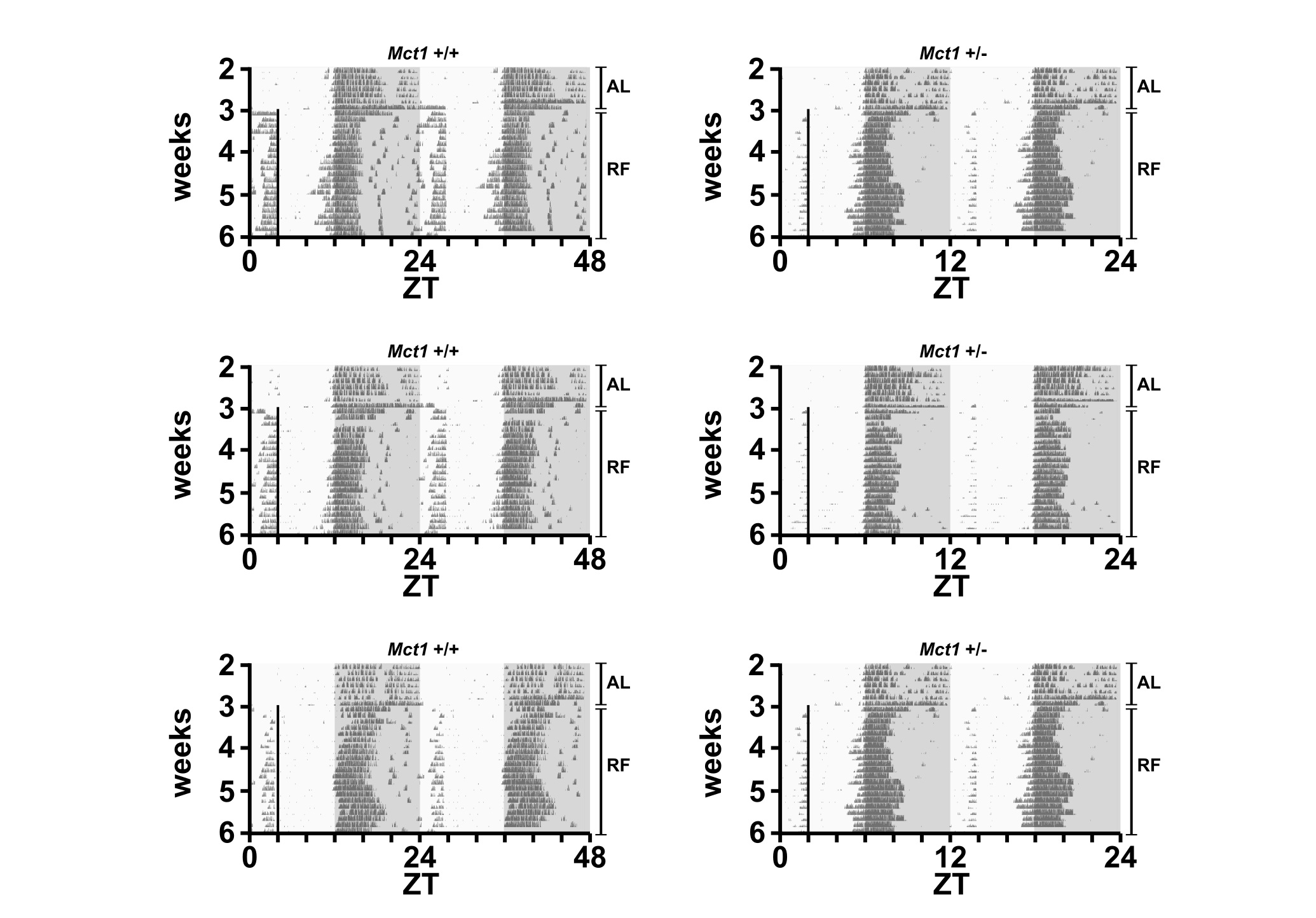

Supplement: Supplementary Figure 1 — Examples of double-plotted wheel-running actograms of Mct1+/+ (left panel) and Mct1+/– mice (right panel) under ad libitum (AL) and daytime-restricted feeding (RF) conditions. The vertical line at ZT4 indicates the time of food access. [file Image_1.JPEG]

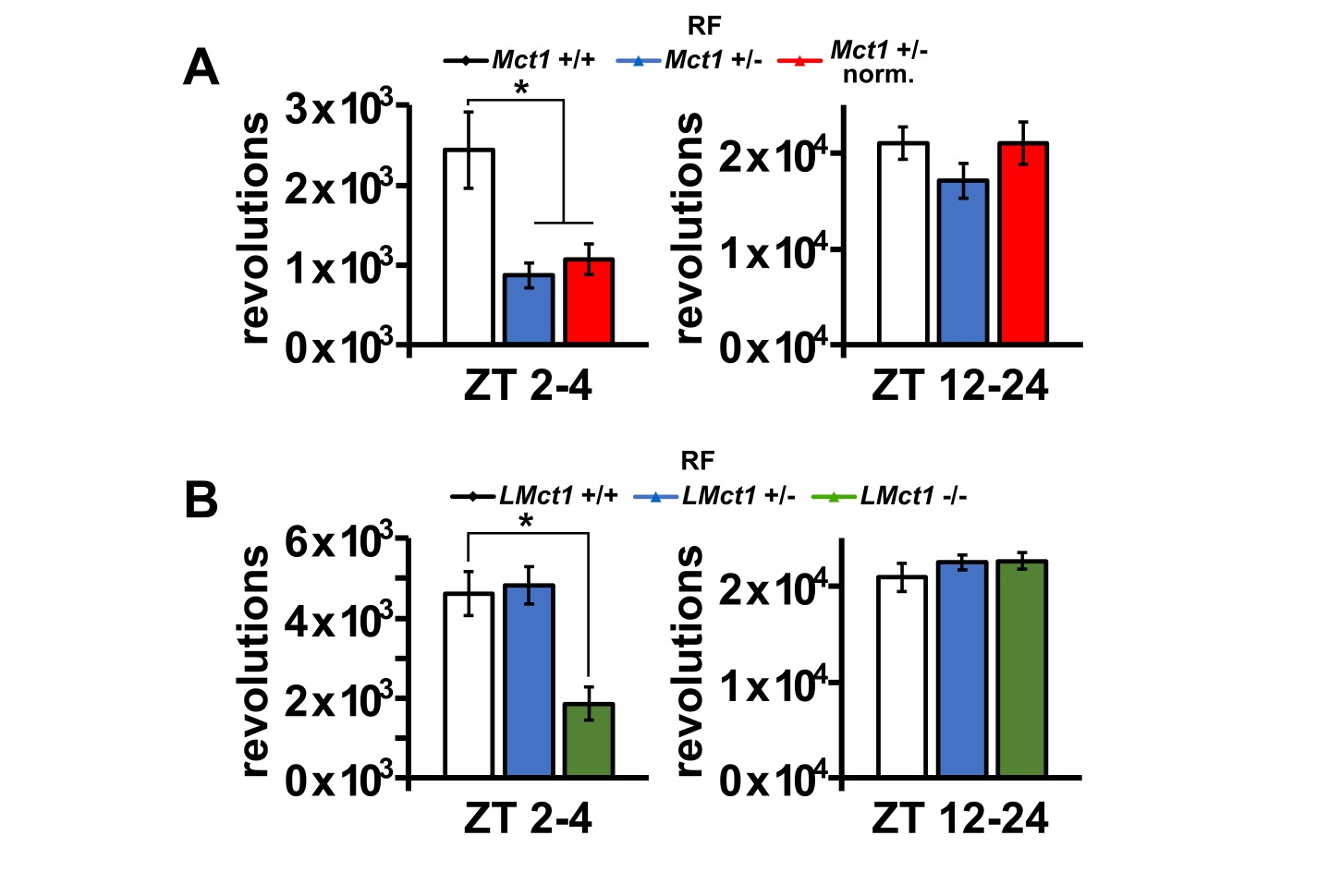

Supplement: Supplementary Figure 2 — (A) Graphical representation of average daily quantified wheel revolutions of Mct1+/+ and Mct1+/– haploinsufficient mice during ZT 2–4 (left panel) and ZT 12–24 (right panel) of their last week of RF. (B) Graphical representation of average daily quantified wheel revolutions of LMct1–/– mice and their controls during ZT 2–4 (left panel) and ZT 12–24 (right panel) of their last week of RF. The quantification includes the time-points that limit the intervals of quantification (closed intervals). The group comparison of non-normalized and normalized data vs. the control group was performed with the 2-tailed Student’s t-test (n = 4–15, ∗p = 0.02 and 0.03, respectively). [file Image_2.JPEG]

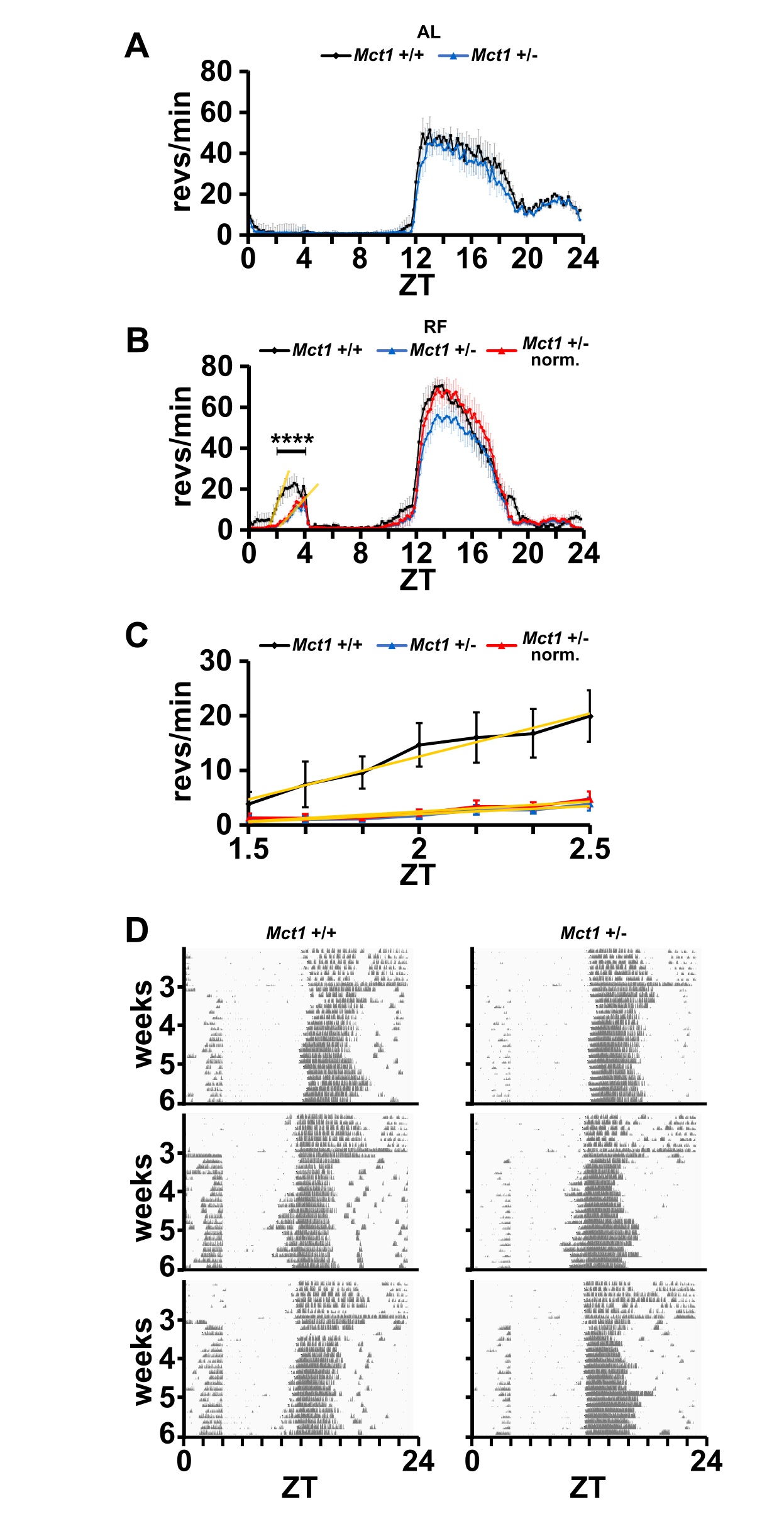

Supplement: Supplementary Figure 3 — Quantified wheel-running activity plots under AL (A) and the last week of RF (B), as in Figure 2, but with 10 min resolution. FAA was compared between ZT2 and ZT4 with a 2-way ANOVA (n = 13–15, ****p = 7.45 × 10^–14^, F = 60.62). The slope of rise in activity before feeding time under RF conditions (yellow line) is significantly flatter in Mct1+/– compared to control animals. (C) Analysis of the onset of activity by performing linear regression on time points from ZT 1.5 to ZT 2.5 (13 measurements) revealed that the control group had an increase of activity of 26 revolutions of the wheel per 10 min, while the Mct1+/– group had an increase of 5, and the Mct1+/– normalized 6 revolutions per 10 min, with R-squared values of 0.96, 0.89, and 0.89, respectively. Error bars represent the standard error of mean. (D) Representative actograms of Mct1+/+ and Mct1+/– mice show that the onset of FAA in the control group is comparable between mice of the group, and it is also robust for each individual mouse, with predictable onsets each day. The mice from the haploinsufficient group show that the FAA onset is different on each day for one single mouse and also within the group. [file Image_3.JPEG]

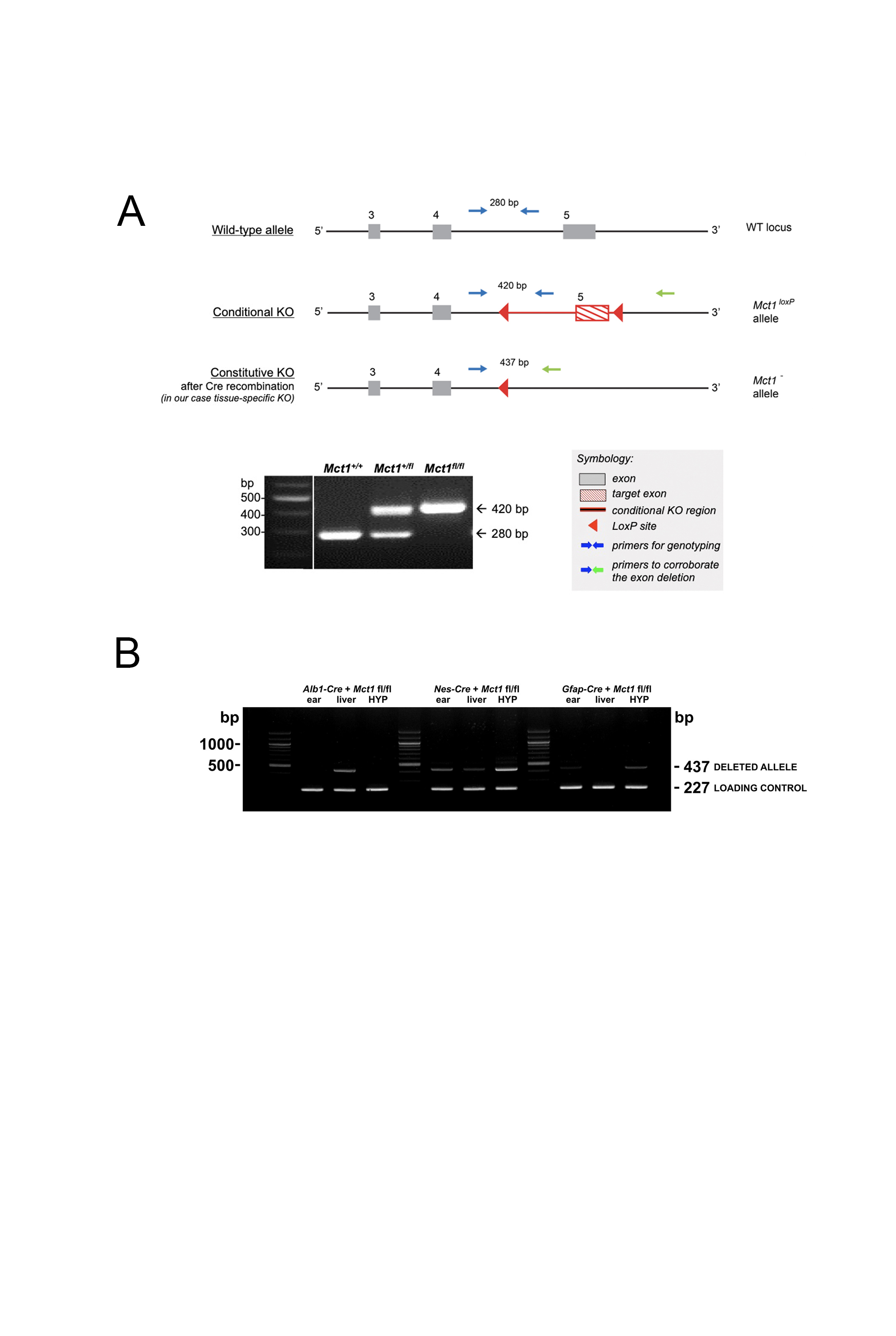

Supplement: Supplementary Figure 4 — Generation of tissue-specific Mct1 KO mice. (A) Top panel: Diagram of the Mct1 wild-type allele (Mct1+/+), conditional KO allele with LoxP sites flanking exon 5 (Mct1fl/fl), and the constitutive KO (Mct1–/–) allele after Cre recombination. Bottom panel: Genotyping results of the corresponding wild-type, heterozygous and homozygous Mct1 floxed mice. The band at 280 bp refers to the wild-type allele, and the band at 420 bp indicates the LoxP insertion. (B) Genotyping of LMct1–/–, NMct1–/–, and GMct1–/– mice reveals a deleted allele at 437 bp in the liver tissue of the Alb1-Cre+ Mct1fl/fl animal and an absence of deleted alleles in the ear biopsy (used for initial genotyping) and the hypothalamus (HYP). In the case of the Nes-Cre driver, the strongest recombined band is noticed in the HYP, but expectedly a weak band could also be detected in the ear and liver tissue. The Gfap-Cre driver shows higher specificity, with recombination detected in the HYP and a weak band in the ear biopsy. [file Image_4.JPEG]

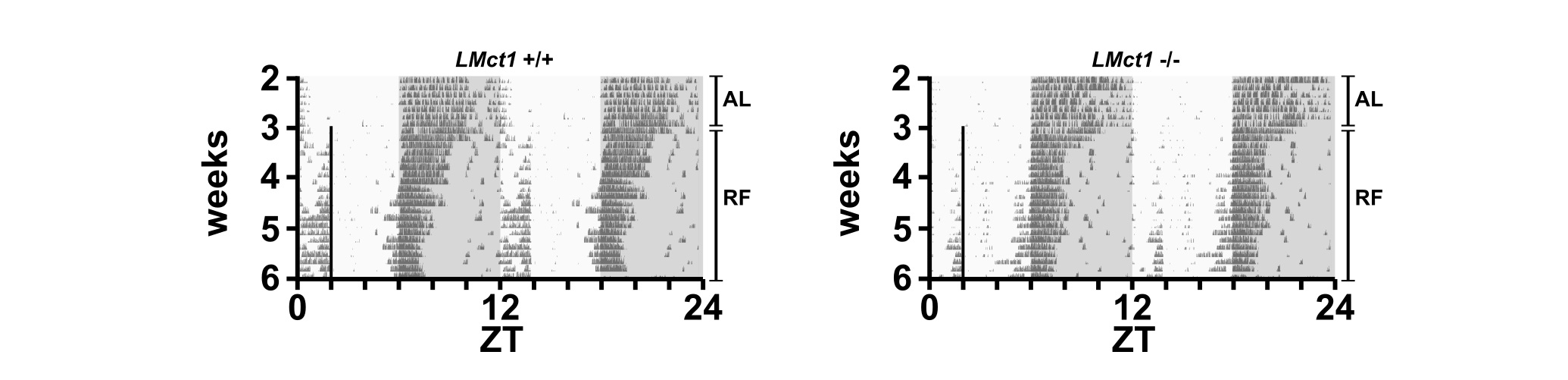

Supplement: Supplementary Figure 5 — Examples of double-plotted wheel-running actograms of LMct1+/+ (left panel) and LMct1–/– mice (right panel) under ad libitum (AL) and daytime-restricted feeding (RF) conditions. The vertical line at ZT4 indicates the time of food access. [file Image_5.JPEG]

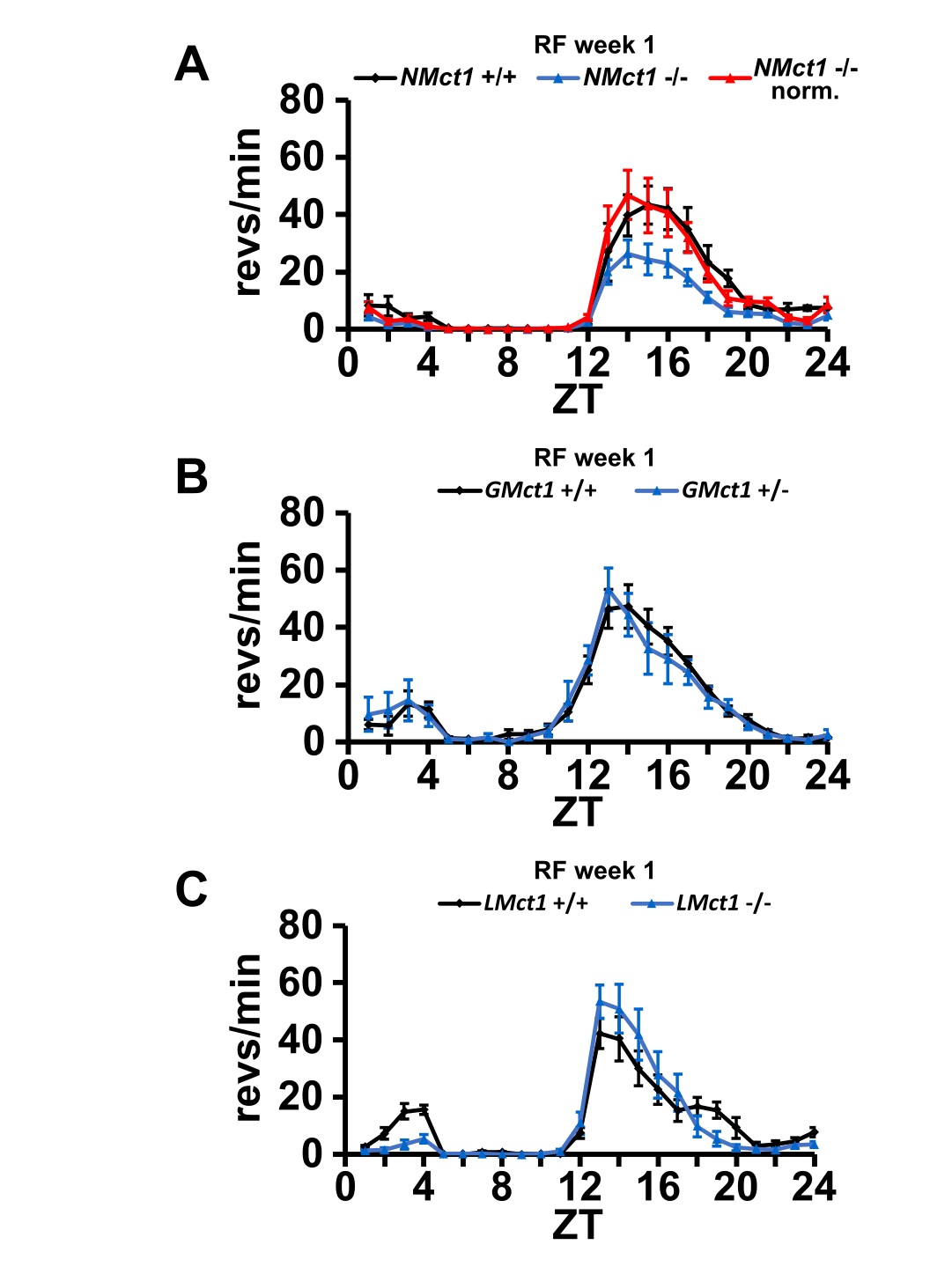

Supplement: Supplementary Figure 6 — Activity profile of first week of RF for (A) neuronal, (B) glial, and (C) liver homozygous Mct1 KO mice vs. corresponding controls shows that neuronal and glial Mct1 KO mice show same patterns of adaptation to RF as their corresponding controls, whereas the adaptation of LMct1–/– mice is already affected in the first week of RF. [file Image_6.JPEG]

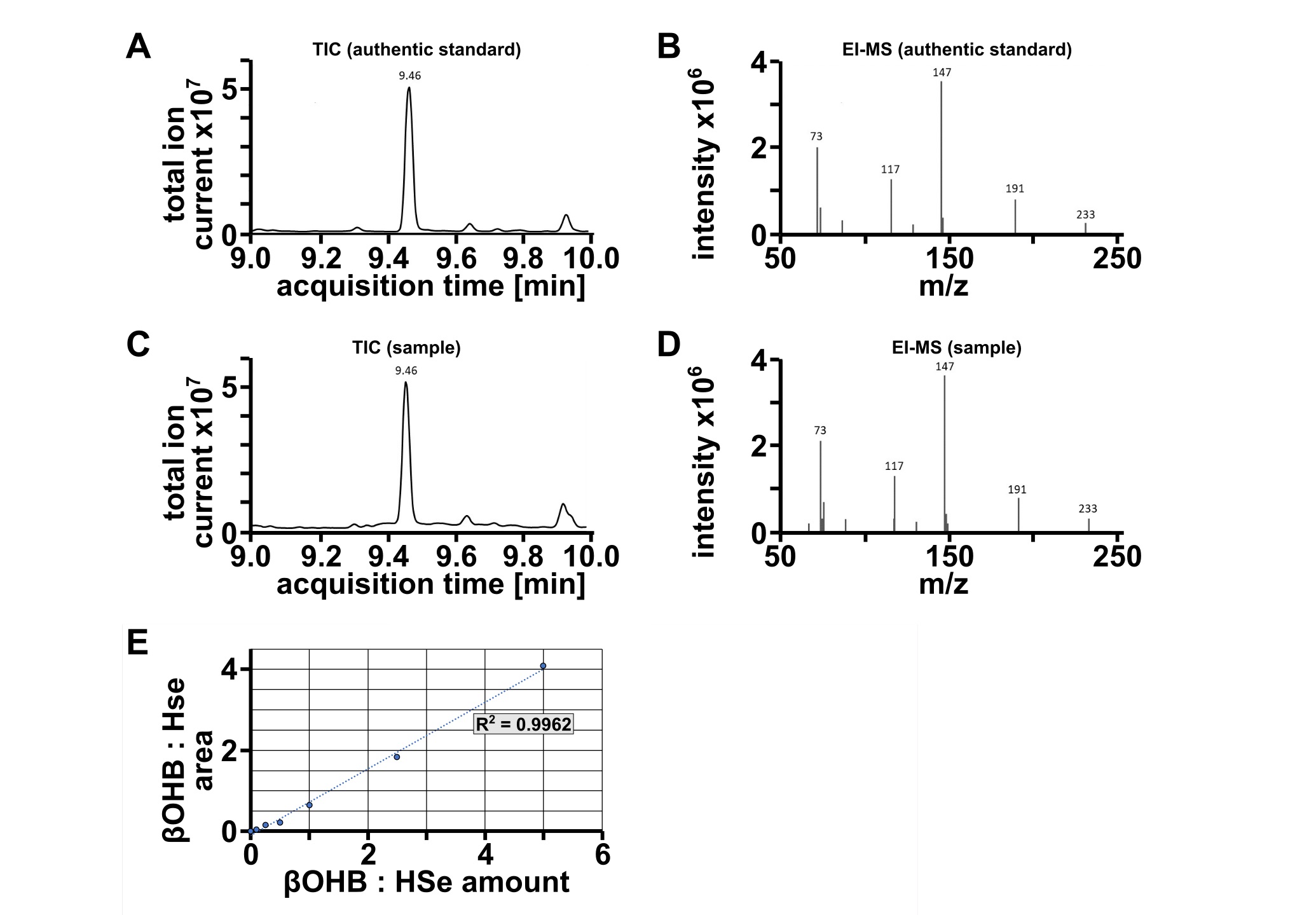

Supplement: Supplementary Figure 7 — Identification of βOHB was done based on (A) retention time and (B) EI-MS fragments of an authentic standard. βOHB in our sample was eluted (C) at the expected retention time of 9.46 min and (D) showed fragments identical to those of the authentic standard. (E) Quantification was based on a 6-point calibration curve. [file Image_7.JPEG]
