# Supplementary material for: The Hepatic Monocarboxylate Transporter 1 (MCT1) Contributes to the Regulation of Food Anticipation in Mice
Source: Front Physiol. 2021 Apr 14;12:665476. doi: 10.3389/fphys.2021.665476 (PMC8079775; doi:10.3389/fphys.2021.665476)
Supplement: Supplementary Table 1 — Quantifications of total wheel revolutions between ZT 2 and 4, as well as between ZT 12 and 24 for each group of animals. The quantification includes the time-points that limit the intervals of quantification (closed intervals). The group comparison of non-normalized and normalized data vs. the control group was performed with the 2-tailed Student’s t-test (n = 4–15). [file Table_1.DOCX]

**Supplemental Table 1**

|  | **Restricted feeding wheel revolutions ZT 2-4 and 12-24 (area under the curve)** | | | | | | | |
| --- | --- | --- | --- | --- | --- | --- | --- | --- |
|  |  |  |  |  |  |  |  |  |
|  | average | SEM | average | SEM | norm. avg. | norm. SEM | p-value | p-value |
| ZT | Mct1 +/+ | Mct1 +/+ | Mct1 +/- | Mct1 +/- | Mct1 +/- | Mct1 +/- | vs. raw | vs. norm. |
| 2-4 | 2436.61 | 478.23 | 872.52 | 158.07 | 1073.82 | 194.54 | 0.01 | 0.02 |
| 12-24 | 21052.85 | 1663.97 | 17106.19 | 1807.28 | 21052.85 | 2224.25 | 0.12 | 1.00 |
|  |  |  |  |  |  |  |  |  |
|  | average | SEM | average | SEM | norm. avg. | norm. SEM | p-value | p-value |
| ZT | NMct1 +/+ | NMct1 +/+ | NMct1 +/- | NMct1 +/- | / | / | vs. raw | vs. norm. |
| 2-4 | 3678.98 | 937.79 | 3583.30 | 666.62 | / | / | 0.93 | / |
| 12-24 | 18008.93 | 1265.60 | 15664.75 | 1686.09 | / | / | 0.33 | / |
|  |  |  |  |  |  |  |  |  |
|  | average | SEM | average | SEM | norm. avg. | norm. SEM | p-value | p-value |
| ZT | NMct1 +/+ | NMct1 +/+ | NMct1 -/- | NMct1 -/- | NMct1 -/- | NMct1 -/- | vs. raw | vs. norm. |
| 2-4 | 2216.80 | 616.94 | 1198.50 | 217.72 | 1924.34 | 349.58 | 0.15 | 0.69 |
| 12-24 | 15281.70 | 1925.92 | 9517.60 | 1049.08 | 15281.70 | 1684.42 | 0.03 | 1.00 |
|  |  |  |  |  |  |  |  |  |
|  | average | SEM | average | SEM | norm. avg. | norm. SEM | p-value | p-value |
| ZT | GMct1 +/+ | GMct1 +/+ | GMct1 +/- | GMct1 +/- | / | / | vs. raw | vs. norm. |
| 2-4 | 3915.70 | 488.58 | 4654.20 | 1000.38 | / | / | 0.52 | / |
| 12-24 | 18852.80 | 2980.93 | 15716.40 | 2203.25 | / | / | 0.42 | / |
|  |  |  |  |  |  |  |  |  |
|  | average | SEM | average | SEM | norm. avg. | norm. SEM | p-value | p-value |
| ZT | GMct1 +/+ | GMct1 +/+ | GMct1 -/- | GMct1 -/- | GMct1 -/- | GMct1 -/- | vs. raw | vs. norm. |
| 2-4 | 5366.16 | 1160.10 | 2713.95 | 764.98 | 3576.88 | 1008.22 | 0.12 | 0.30 |
| 12-24 | 22835.04 | 1565.50 | 17326.05 | 2756.65 | 22835.04 | 3633.15 | 0.11 | 1.00 |
|  |  |  |  |  |  |  |  |  |
|  | average | SEM | average | SEM | average | SEM | p-value | p-value |
| ZT | LMct1 +/+ | LMct1 +/+ | LMct1 +/- | LMct1 +/- | LMct1 -/- | LMct1 -/- | vs. Mct1 +/- | vs. Mct1 -/- |
| 2-4 | 4619.12 | 551.73 | 4816.35 | 464.20 | 1856.40 | 415.75 | 0.87 | 0.03 |
| 12-24 | 20907.28 | 1445.11 | 22467.45 | 772.08 | 22632.30 | 859.60 | 0.60 | 0.57 |
|  |  |  |  |  |  |  |  |  |
| The p-values were calculated with the Student's two-tailed t-test. | | | | |  |  |  |  |
| Normalisation was done based on comparison of night-time activity (ZT 12-24). | | | | | |  |  |  |
