# Supplementary material for: The Hepatic Monocarboxylate Transporter 1 (MCT1) Contributes to the Regulation of Food Anticipation in Mice
Source: Front Physiol. 2021 Apr 14;12:665476. doi: 10.3389/fphys.2021.665476 (PMC8079775; doi:10.3389/fphys.2021.665476)
Supplement: Supplementary Table 2 — Results of statistical analysis of comparison of FAA ZT2-4 using 2-way ANOVA. [file Table_2.DOCX]

Haploinsufficient mice vs. controls, 1 h bin, not normalized

Response: Value

Df Sum Sq Mean Sq F value Pr(>F)

ZT 1 138.2 138.15 1.2674 0.2636

Group 1 1993.2 1993.23 18.2849 5.22e-05 ***

ZT:Group 1 61.0 60.95 0.5591 0.4568

Residuals 80 8720.8 109.01

---

Signif. codes: 0 ‘***’ 0.001 ‘**’ 0.01 ‘*’ 0.05 ‘.’ 0.1 ‘ ’ 1

Haploinsufficient mice vs. controls, 1 h bin, normalized

Response: Value_norm

Df Sum Sq Mean Sq F value Pr(>F)

ZT 1 197.0 196.99 1.7211 0.1932985

Group 1 1512.8 1512.80 13.2179 0.0004888 ***

ZT:Group 1 105.2 105.22 0.9193 0.3405371

Residuals 80 9156.1 114.45

---

Signif. codes: 0 ‘***’ 0.001 ‘**’ 0.01 ‘*’ 0.05 ‘.’ 0.1 ‘ ’ 1

Haploinsufficient mice vs. controls, 10 min bin, not normalized

Response: Value

Df Sum Sq Mean Sq F value Pr(>F)

ZT 1 1153 1152.9 7.4727 0.006573 **

Group 1 13106 13105.6 84.9456 < 2.2e-16 ***

ZT:Group 1 538 538.2 3.4886 0.062604 .

Residuals 360 55542 154.3

---

Signif. codes: 0 ‘***’ 0.001 ‘**’ 0.01 ‘*’ 0.05 ‘.’ 0.1 ‘ ’ 1

Haploinsufficient mice vs. controls, 10 min bin, normalized

Response: Value_norm

Df Sum Sq Mean Sq F value Pr(>F)

ZT 1 1618 1617.5 9.8349 0.001853 **

Group 1 9969 9969.2 60.6154 7.454e-14 ***

ZT:Group 1 896 895.7 5.4461 0.020162 *

Residuals 360 59208 164.5

---

Signif. codes: 0 ‘***’ 0.001 ‘**’ 0.01 ‘*’ 0.05 ‘.’ 0.1 ‘ ’ 1

Haploinsufficient mice vs. controls, general activity, 1 h bin, not normalized

Response: Value

Df Sum Sq Mean Sq F value Pr(>F)

ZT 1 55.0 55.03 0.3725 0.54459

Group 1 1037.5 1037.55 7.0229 0.01093 *

ZT:Group 1 0.0 0.00 0.0000 0.99737

Residuals 47 6943.7 147.74

---

Signif. codes: 0 ‘***’ 0.001 ‘**’ 0.01 ‘*’ 0.05 ‘.’ 0.1 ‘ ’ 1

NMct1+/- vs. controls, 1 h bin, not normalized

Response: Value

Df Sum Sq Mean Sq F value Pr(>F)

ZT 1 147.1 147.111 0.6348 0.4290

Group 1 4.1 4.068 0.0176 0.8951

ZT:Group 1 16.1 16.131 0.0696 0.7929

Residuals 56 12977.0 231.732

NMct1-/- vs. controls, 1 h bin, not normalized

Response: Value

Df Sum Sq Mean Sq F value Pr(>F)

ZT 1 3.47 3.466 0.0576 0.81192

Group 1 288.04 288.037 4.7843 0.03614 *

ZT:Group 1 15.75 15.746 0.2615 0.61257

Residuals 32 1926.56 60.205

---

Signif. codes: 0 ‘***’ 0.001 ‘**’ 0.01 ‘*’ 0.05 ‘.’ 0.1 ‘ ’ 1

NMct1-/- vs. controls, 1 h bin, normalized

Response: Value_norm

Df Sum Sq Mean Sq F value Pr(>F)

ZT 1 13.15 13.155 0.1755 0.6781

Group 1 23.76 23.759 0.3169 0.5774

ZT:Group 1 32.87 32.873 0.4385 0.5126

Residuals 32 2398.96 74.967

GMct1+/- vs. controls, 1 h bin, not normalized

Response: Value

Df Sum Sq Mean Sq F value Pr(>F)

ZT 1 112.3 112.28 0.5796 0.4521

Group 1 151.5 151.50 0.7820 0.3831

ZT:Group 1 133.0 132.96 0.6863 0.4135

Residuals 32 6199.3 193.73

GMct1-/- vs. controls, 1 h bin, not normalized

Response: Value

Df Sum Sq Mean Sq F value Pr(>F)

ZT 1 112.4 112.40 0.4427 0.51244

Group 1 1447.4 1447.37 5.7005 0.02556 *

ZT:Group 1 190.8 190.78 0.7514 0.39499

Residuals 23 5839.7 253.90

---

Signif. codes: 0 ‘***’ 0.001 ‘**’ 0.01 ‘*’ 0.05 ‘.’ 0.1 ‘ ’ 1

GMct1-/- vs. controls, 1 h bin, normalized

Response: Value_norm

Df Sum Sq Mean Sq F value Pr(>F)

ZT 1 333.0 333.02 0.8487 0.3665

Group 1 151.9 151.93 0.3872 0.5399

ZT:Group 1 500.1 500.06 1.2743 0.2706

Residuals 23 9025.3 392.40

LMct1+/- vs. controls, 1 h bin, not normalized

Response: Value

Df Sum Sq Mean Sq F value Pr(>F)

ZT 1 707.2 707.19 3.0723 0.08542 .

Group 1 11.4 11.37 0.0494 0.82494

ZT:Group 1 78.6 78.59 0.3414 0.56150

Residuals 53 12199.7 230.18

---

Signif. codes: 0 ‘***’ 0.001 ‘**’ 0.01 ‘*’ 0.05 ‘.’ 0.1 ‘ ’ 1

LMct1-/- vs. controls, 1 h bin, not normalized

Df Sum Sq Mean Sq F value Pr(>F)

ZT 1 578.3 578.29 2.7739 0.101713

Group 1 2231.8 2231.76 10.7051 0.001883 **

ZT:Group 1 15.5 15.49 0.0743 0.786221

Residuals 53 11049.2 208.48

---

Signif. codes: 0 ‘***’ 0.001 ‘**’ 0.01 ‘*’ 0.05 ‘.’ 0.1 ‘ ’ 1
